# *Sphingomonas* Relies on Chemotaxis to Degrade Polycyclic Aromatic Hydrocarbons and Maintain Dominance in Coking Sites

**DOI:** 10.3390/microorganisms10061109

**Published:** 2022-05-27

**Authors:** Meng Zhou, Zishu Liu, Jiaqi Wang, Yuxiang Zhao, Baolan Hu

**Affiliations:** 1 Department of Environmental Engineering, College of Environmental & Resources Sciences, Zhejiang University, Hangzhou 310058, China; 11914036@zju.edu.cn (M.Z.); liuzishu@zju.edu.cn (Z.L.); tudou@zju.edu.cn (J.W.); 21814091@zju.edu.cn (Y.Z.); 2 Zhejiang Province Key Laboratory for Water Pollution Control and Environmental Safety, Hangzhou 310058, China; 3 Key Laboratory of Environment Remediation and Ecological Health, Ministry of Education, College of Environmental Resource Sciences, Zhejiang University, Hangzhou 310058, China

**Keywords:** soil, PAH degradation, *Sphingomonas*, microorganisms, metagenomics

## Abstract

Polycyclic aromatic hydrocarbons (PAHs) are organic pollutants widely present in industrial sites. Microbial degradation is an effective method of removing PAHs. The identification of microorganisms that have important ecological functions at the site is of great significance for PAH removal. We collected soil samples at three depths in the range of 0–100 cm at 70-day intervals at the coking site and explored the degradation of PAHs. We combined molecular ecology networking, metagenomics, and genome assembly to search for microorganisms that persist, dominate, and affect the microbial community construction in the degradation process and analyzed their adaptation strategies. The results showed that 15.78 mg/kg of PAHs naturally decayed, and 13.33 mg/kg of PAHs migrated from 30–100 cm to 0–30 cm in the soil. *Sphingomonas*, which occupied a niche advantage, was both the core and keystone microorganism, and its spatial distribution pattern and temporal change dynamics were consistent with those of PAHs. We assembled the genome of *Sphingomonas* sp., revealing its multiple potential for degrading PAHs and other pollutants. Additionally, flagellar assembly and bacterial chemotaxis genes ranked high in the assembled genome of *Sphingomonas* sp., which might help it obtain a competitive advantage in the soil. The findings underscored the strategy of *Sphingomonas* to maintain dominance, enriched the understanding of PAH-degrading microorganisms in site soil, and provided references for the remediation of PAHs.

## 1. Introduction

PAHs are widely distributed persistent organic pollutants. The U.S. Environmental Protection Agency identified sixteen PAHs with two to six benzene rings as priority pollutants [1]. Some low-molecular-weight PAHs (LMW-PAHs) are highly toxic, and most high-molecular-weight PAHs (HMW-PAHs) are mutagenic and potentially carcinogenic [2].

PAHs are commonly found in sites associated with the oil production, gas production, and wood processing industries, derived from coal tar, petroleum sludge, creosote, and wood preservative wastes [3]. PAHs are everywhere, and their ultimate destination is the soil. In fact, 90% of the global environmental PAH load exists in soil [4]. Considering the potential toxicity of PAHs, risk exposure level, and site construction requirements, the remediation of PAH-contaminated-site soils is very important [3]. The concentration of PAHs in soils has increased over the past 30 years, especially in industrialized regions of the world, and it will continue to increase over the next 5 years or more due to increased anthropogenic emissions [3]. To date, published reports have mainly focused on the PAH concentrations in contaminated soils. However, a large number of industrial contaminated sites remain to be explored due to the inaccessibility of abandoned industrial contaminated sites to researchers due to the legal and liability issues related to the transport and storage of contaminated soil.

Various techniques, including biodegradation and photochemical degradation, have been used to remove PAHs. However, biodegradation is the most dominant and feasible bioremediation technology for PAH removal [5]. Several different bacterial genera are known for their powerful PAH catabolism, especially LMW-PAHs, including *Mycobacterium*, *Pseudomonas*, *Sphingomonas*, and *Sphingobacter* [6]. To date, the remediation of PAHs by single strains or compound strains has been intensively studied. A group of highly efficient bacterial consortia composed of *Sphingosine* (58.57–72.40%) and *Pseudomonas* (25.93–39.75%), which can utilize phenanthrene as the sole carbon source and can also degrade naphthalene, achieved a 100% PAH removal rate within 5 days [7].

The degradation of contaminants by microorganisms first requires exposure to the contaminant, so sensing the presence of the contaminant and coming into contact with it through movement may be an important step. Chemotaxis is the widespread ability of motile microorganisms to direct their movement along chemical gradients. The use of flagella for bacterial motility is the hallmark strategy of chemotaxis [8]. The rotation of a flagellar filament is driven at its base by a reversible rotary motor. The flagellar filament, the motor, and the components that connect them (the rod and the hook) form a supramolecular complex called a ‘flagellum’ [9]. Motility and chemotaxis were thought to be among the important autecological properties for PAH degradation by bacteria in contaminated soils [10]. Chemotaxis was evaluated as a bioavailability-promoting trait in PAH-degrading bacteria from the rhizosphere for the first time in 2003. Chemotaxis was considered to be a relevant mobilizing factor for PAH-degrading rhizosphere bacteria [11]. The co-transport of PAHs by motile microorganisms leads to enhanced mass transfer under diffusive conditions. The effective diffusivity of *T. pyriformis*, determined by video imaging microscopy, was found to exceed the molecular diffusivities of PAHs up to four-fold [12]. Metagenomics has been widely used to study microbial communities, which strongly support the exploration of the functional potential and metabolic pathways of PAH-degrading microorganisms [13]. *Pseudomonas putida *B6-2 is a broad-spectrum aromatic polycyclic degrading microorganism capable of resisting highly toxic organic solvents, and metagenomics indicated that *Pseudomonas putida *B6-2 may have the metabolic pathway and chemotaxis of PAHs. Genomic evidence suggests that bacterial chemotaxis, efflux pumps, and transport proteins make B6-2 a potential candidate for the remediation of highly polluted environments [14]. The draft genome sequence of a *Pseudomonas* SMT-1 strain isolated from a landfill provides useful resources for elucidating the molecular mechanism of fluorene catabolism [15]. Molecular ecology network analysis helps to clarify complex interactions between PAH-degrading microorganisms and other microorganisms in the community [16]. Combining molecular ecology network analysis with metagenomics helps to identify microorganisms that play important ecological functions in PAH-contaminated soils and elucidate their functional potential and metabolic pathways. There has been excellent work combining metagenomics and molecular ecology network analysis to clarify the degradation and metabolic pathways of PAHs by in situ rhizosphere microorganisms [17]. However, the identification of key microorganisms that have an important impact on the microbial community structure and their ecological functions in PAH-contaminated sites still needs further exploration.

In order to grasp the migration and degradation of 16 PAHs at coking sites, as well as to reveal the dominant, core, and keystone microorganisms during the degradation process, we collected soils from different depths (0–10 cm top layer, 10–30 cm middle layer, and 30–100 cm bottom layer) at 70-day intervals at a coking site. Molecular ecology network analysis, metagenomics, and genome assembly were used to locate the core microorganisms that persist in the site soil, the dominant microorganisms that occupy niche advantages, and the keystone microorganisms that have an important impact on the construction of microbial communities. The correlation between key microorganisms and PAH migration and degradation was analyzed, and the strategies of key microorganisms to adapt to environmental pollution and maintain the dominant position were explored.

## 2. Materials and Methods

### 2.1. Collection of Soil Samples

The soil samples were collected at a metallurgical plant that had a production history of 60 years. On 7 April 2020 and 17 June 2020, soil samples of the 0–10 cm, 10–30 cm, and 30–100 cm layers were collected in the PAH-polluted area (30°36′ N, 120°16′ E) in Zhejiang Province, China. The soil samples were collected by the five-point sampling method, and were thoroughly mixed. A total of 6 soil samples were obtained after mixing. Each sample was divided into two parts, one was stored in a refrigerator at 4 °C for physical and chemical analysis, and the other was stored in a refrigerator at −20 °C for DNA extraction.

### 2.2. Determination of 16 PAHs

Sixteen PAHs were detected by the HJ 784-2016 method (Determination of PAHs in Soil and Sediment by High Performance Liquid Chromatography) using GC-MS (TRACE 1300-ISQ DQ300, HLJC-115, Waltham, MA, USA). Three replicates were tested for each sample. The recoveries of the spiked standard were 91.3–114%. The detection limits for the samples are listed in Table S1.

### 2.3. DNA Extraction, 16S rRNA Gene Amplicon Sequencing, and Analysis

A Power Soil DNA Isolation Kit (MoBio Laboratories Inc., Carlsbad, CA, USA) was used to extract DNA from soil samples. The extracted DNA was tested for concentration using a NanoDrop 2000 spectrophotometer (Thermo Fisher Scientific, Waltham, MA, USA).

Library construction and sequencing were carried out using Illumina MiSeq [18]. DNA amplification was performed using the universal 16S primer 338F/806R carrying brocade (338F 5′-3′ ACTCCTACGGGAGGCAGCAG, 806 R 5′-3′ GGACTACHVGGGTWTCTAAT) [19,20]. The PCR system was: 12.5 μL 2 × TB Green Premix Ex Tap (TakaRa Bio Inc., Kusatsu, Japan), 1 μL forward primer (10 μmol/L), 1 μL reverse primer (10 μmol/L), 1 μL DNA (1–10 ng), and 9.5 μL RNAse-free water (TakaRa Bio Inc., Japan). The PCR program included denaturation at 94 °C for 3 min, 35 cycles (denaturation at 94 °C for 40 s, annealing at 55 °C for 30 s, and extension at 72 °C for 40 s), and extension at 72 °C for 8 min. The PCR products were purified using the Axygen PCR purification kit (Axygen, San Francisco, CA, USA). The samples were sequenced and subjected to subsequent analysis by Guangdong MAGIGENE Technology Co., Ltd. (MAGIGENE, Guangzhou, China). The raw reads obtained by sequencing were cut and filtered using the QIIME software package (www.qiime.org, accessed date, 16 July 2020) to obtain valid data (Clean reads), which were spliced into Tags by end matching and further filtered to obtain the target fragments (Clean tags). The Tags were clustered into operational taxonomic units (OTU) with 97% similarity, and annotation was performed using the Ribosomal Database Project (RDP) Classifier to obtain the community composition information of each sample. A total of 6 soil samples were sequenced. The raw data from the sequencing were uploaded to the NCBI database: PRJNA823401. The results of read filtering in sequencing data analysis can be found in Table S3. Gel electrophoresis images and the concentrations of PCR products can be found in Figures S3 and S4, and Tables S5 and S6.

### 2.4. Screening for Keystone Species, Core Species, and Dominant Species

Keystone species, core species, and dominant species have important impacts on the microbial community’s structure and its ecological functions. Keystone species refer to species that have a huge impact on the community structure in the habitat disproportionate to their abundance, play a crucial role in maintaining community structure, and significantly affect other microorganisms in the ecosystem. Once the keystone species are removed from the habitat, the community structure of the entire habitat will change dramatically [21]. Core species are species that persist in the habitat, and correspond to the species that temporarily exist in the habitat (transient species). The relative abundance of a microorganism in the habitat and its frequency of occurrence in different samples can be used to determine whether the microorganism is a core species in this habitat [22,23,24]. The dominant species are the species with an ecological competitive advantage in the habitat.

Keystone species in the habitat were identified by constructing genus-level molecular ecology networks to explore the interactions between different microorganisms. The microbial genera with a relative abundance of less than 0.1% in the sample were excluded, and an initial species network correlation matrix based on Spearman correlation analysis was constructed using the WGCNA package in the R language (V3.6.0) [25]. Data with Spearman correlation coefficients greater than 0.7 and *p* values less than 0.05 were used to construct the molecular ecology network, and the derived random matrix was visualized using Gephi software (V0.9.2) [26].

The screening conditions of core species varied in different studies. According to available reports, the core species screening criteria were set at the genus level as follows: (1) mean relative abundance in all samples >0.1% [27,28]; and (2) occurrence frequency >80% in all samples [29].

### 2.5. Metagenomic Sequencing

After the concentration of the soil DNA was qualified, the buffer was added, and an ultrasonic breaker was used for random interruption. The short fragments of DNA obtained after the interruption were used for library construction. For the library qualified by Agilent 2100 Bioanalyzer (Agilent, Santa Clara, CA, USA) quality inspection, the Illumina HiSeq 2500 high-throughput sequencing platform was used for PE150 sequencing to obtain raw sequenced data [18].

Trimmomactic software [30] was used to control the quality of the raw data and perform metagenomic assembly on each sample. MEGAHIT [31] was used to splice the data after quality control. The sequence fragments with a splicing length of more than 500 bp were screened for subsequent analysis. Linclust software [32] was used for gene clustering and de-redundancy. The longest sequence of each cluster was selected as the representative sequence to obtain non-redundant gene protein sequences [33]. The functional annotation information was obtained by comparing gene protein sequences with databases, such as KEGG, eggNOG, CAZyme, and CARD.

### 2.6. Genome Assembly

Whole-genome sequencing (WGS) was performed on the Illumina MiSeq and Pacbio Sequel platforms. GeneMarkS (http://topaz.gatech.edu/GeneMark/, accessed date, 18 June 2021) was used to predict the entire gene sequence. For the functional annotation of protein-coding genes, sequence alignments were performed using diamond (http://github.com/bbuchfink/diamond, accessed date, 18 June 2021). The databases used for sequence alignment were NR (ftp://ftp.ncbi.nih.gov/blast/db/, accessed date, 18 June 2021) and Swiss-Prot (http://www.uniprot.org/, accessed date, 18 June 2021). The critical value of sequence alignment was 1 × 10^−6^, and the best hit was selected. The *Sphingomonas* sp. genome sequencing was uploaded to the NCBI database: PRJNA821642.

### 2.7. Data Analysis and Picture Drawing

Prism software (GraphPad Prism 8.4.2) was used for data processing and drawing. Some image materials were obtained from the Figdraw platform (www.figdraw.com, accessed date, 19 March 2022).

## 3. Results and Discussion

### 3.1. Occurrence Patterns, Migration, and Transformation Dynamics of PAHs in Soil at Different Depths in Coking Sites

**Figure 1:**
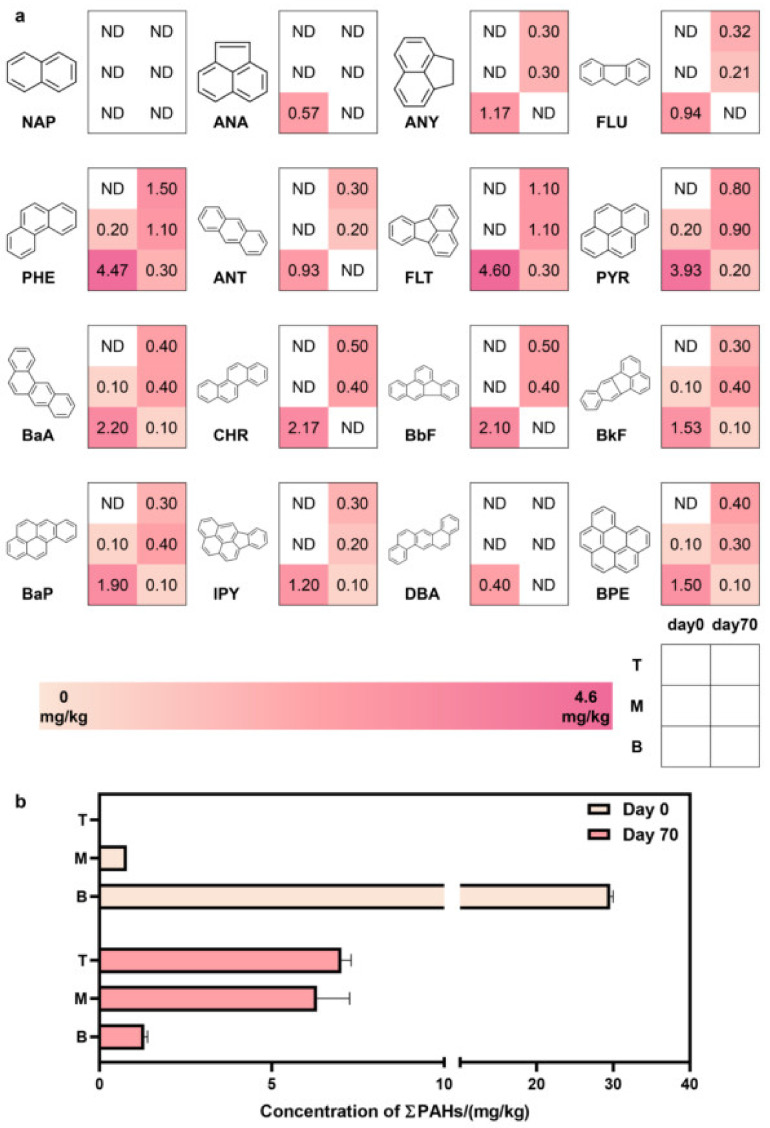
Concentrations of 16 PAHs (**a**) and total PAHs (**b**) in coking site soils sampled at depths of 0–10 cm (T: top layer), 10–30 cm (M: middle layer), and 30–100 cm (B: bottom layer) on Day 0 (in light color) and Day 70 (in dark color). Average concentration per kind of PAH is labeled in the corresponding cell and ND denotes not detected.

The total accounting and monomer determination of 16 PAHs in coking site soil showed that (Figure 1), except for naphthalene, which was not detected at each depth and time, the other 15 PAHs were detected. The PAHs with the lowest concentration were benzo[a, h]anthracene (DBA), and only 0.4 mg/kg was detected in the bottom layer on Day 0; the highest-concentration PAH was phenanthrene (PHE), and 4.47 mg/kg was detected in the bottom layer on Day 0, with an average concentration of 1.26 mg/kg. After 70 days, a total of 15.78 mg/kg of PAHs naturally decayed in the 0–100 cm site soil. The degradation amounts of 15 PAHs, except for naphthalene, were 0.4–2.23 mg/kg, of which benzo[a, h]anthracene and pyrene accounted for 0.4 mg/kg and 2.23 mg/kg, respectively. A total of 13.33 mg/kg PAHs migrated from the bottom layer to the middle and top layers, and the PAH concentrations in the top layer increased from not detected to 7.02 mg/kg.

This migration from the bottom layer to the top layer may lead to more volatilization of PAHs in the top-layer soil, resulting in increased potential human health risks.

Industrial activities related to the production and use of coal tar/asphalt resulted in significant amounts of phenanthrene, fluoranthene, and pyrene. Coke production is usually associated with substances containing anthracene, phenanthrene, and benzo[a]pyrene [3,34]. The study area was located in a coking site, so the concentrations of phenanthrene, fluoranthene, and pyrene ranked the top three among the 16 PAH concentrations, with average concentrations of 1.26 mg/kg, 1.18 mg/kg, and 1.01 mg/kg, respectively.

### 3.2. Dominant, Keystone, and Core Species of Coking Site Soil

**Figure 2:**
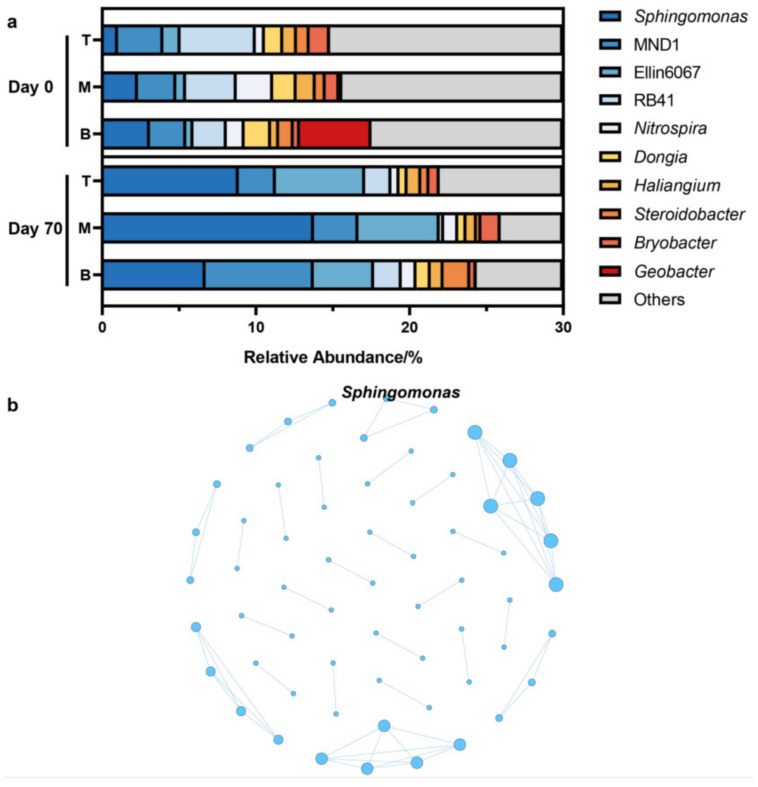
Relative abundance of the top-10 genera in the coking site (T: 0–10 cm; M: 10–30 cm; B: 30–100 cm) (**a**) and genus-level molecular ecology network (**b**).

*Sphingomonas* had the highest average relative abundance of 5.98% among the 507 genera in the soil of the coking site (Figure 2a). In comparison with Day 0, the values of Chao1 decreased by 33.3% after 70 days, which implied a reduction in the microbial species. Meanwhile, the values of the Shannon index dropped to 89.0% of the initial values, meaning a loss of biodiversity when considering the relative abundance per species. As the most abundant genus, the proportion of *Sphingomonas* increased from 2.16% to 9.80% within the time period of 70 days. The decrease in microbial species and homogeneity, combined with the population growth of *Sphingomonas*, indicated that *Sphingomonas* could well adapt to the contaminated environment and gain a competitive advantage in PAH-contaminated sites.

Based on the criteria of an average relative abundance of >0.1% and occurrence frequency of >80%, the core species in the site soil were screened. A total of 64 core species were screened from 507 genera (Table S2). The results showed that *Sphingomonas* persisting in PAH-contaminated sites were the core species in the soil.

Networks are the visualization of potential microbial interactions, in which nodes represent different taxa and edges represent the correlations of the relative abundance among taxa. The molecular ecology network was used to analyze the relationship between microbes in the coking site. Taking a Spearman correlation coefficient r of ≥ 0.7 and *p* value of <0.05 as the standard, and using the BH method to correct the *p* value, the molecular ecology network was constructed. The molecular ecology network had 61 nodes, and the average degree was 1.967. The results showed that *Sphingomonas* participated in the construction of the site’s soil molecular ecology network (Figure 2b), played a crucial role in maintaining the community’s structure, and significantly affected other microorganisms. *Sphingomonas* was the keystone species of the PAH-contaminated site’s soil.

The spatial distribution pattern and temporal variation of *Sphingomonas* were consistent with those of the PAHs. On Day 0, the relative abundance of *Sphingomonas* and the concentration of PAHs gradually increased from the top layer to the bottom layer. On Day 70, with the migration of PAHs from the bottom layer to the middle and top layers, the concentration of PAHs in bottom layer was lower than that in the middle and top layers, and the relative abundance of *Sphingomonas* was also higher in the middle and top layers than that in bottom layer. There have been similar reports that *Sphingomonas* is distributed with the spatial distribution of toxic substances. For example, Isoproturon (IPU), commonly used in farmlands, remains in the soil and migrates to the bottom layer along with surface water and groundwater. The biodegradation of IPU was detected in the soil and a spatial variation in the biodegradation rate of IPU was found. This spatial variation was the result of a combination of microbial degradation (*Sphingomonas* spp.) and soil pH [35].

### 3.3. Metabolic Potential and Survival Strategies of Sphingomonas sp.

**Figure 3:**
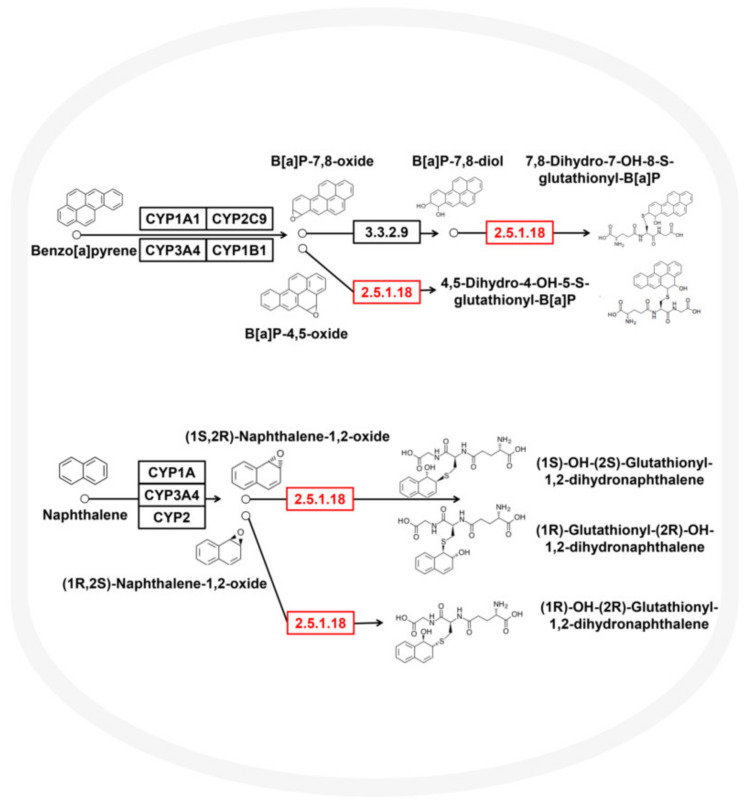
*Sphingomonas* sp. metabolic pathways for the degradation of PAHs.

We obtained the *Sphingomonas* sp. genome with completeness of 98.57% and contamination of 2.61. Circos v0.69 (http://circos.ca/, accessed date, 18 June 2021) software was used to draw the genome circle map (Figure S1). We found that *Sphingomonas* sp. contained 4110 predicted genes. The genome size was 2.52 Mb and the GC content was 67.70%. The gene density was 0.94 genes per kb, and the average gene length was 953.34 bp. Twenty tRNA genes and two sRNA genes were identified in the genome. A variety of genes related to the metabolism of aromatic compounds, PAH metabolism, and P450 metabolism were found in the genome sequence of *Sphingomonas* sp., which is the reason why *Sphingomonas* sp. can effectively degrade PAHs. Fifteen genes were related to the metabolism of cytochrome P450 to xenobiotics, ten genes were related to the degradation of aromatic compounds, and three genes were related to the degradation of PAHs. The genomic data (Figure 3) indicated that *Sphingomonas* sp. possessed a metabolic pathway that mediated naphthalene degradation—(1S,2R)-Naphthalene 1,2-oxide + Glutathione <=> (1S)-Hydroxy-(2S)-glutathionyl-1,2-dihydronaphthalene reaction, (1R,2S)-Naphthalene 1,2-oxide + Glutathione <=> (1R)-Hydroxy-(2R)-glutathionyl-1,2-dihydronaphthalene reaction—and a benzo[a]pyrene degradation pathway—Benzo[a]pyrene-4,5-oxide + Glutathione <=> 4,5-Dihydro-4-hydroxy-5-S-glutathionyl-benzo[a]pyrene reaction, Benzo[a]pyrene-7,8-dihydrodiol + Glutathione <=> 7,8-Dihydro-7-hydroxy-8-S-glutathionyl-benzo[a]pyrene + H_2_O.

**Figure 4:**
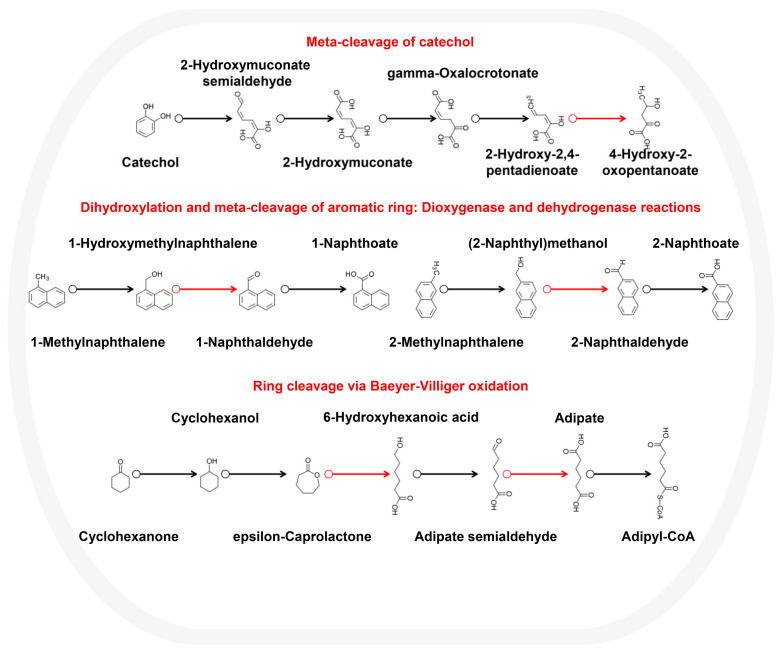
*Sphingomonas* sp. metabolic pathways for the degradation of aromatic compounds.

*Sphingomonas* sp. might participate in a wide range of aromatic catabolism processes, such as the meta-cleavage of the catechol pathway, where the *mhpD* gene confers the function of converting 2-Hydroxy-2,4-pentadienoate to 4-Hydroxy-2-oxopentanoate, and in the dihydroxylation and meta-cleavage of aromatic rings mediated by dioxygenase and dehydrogenase, where the *adpH* gene confers the function of converting 1-Hydroxymethylnaphthalene and (2-Naphthyl)methanol to 1-Naphthaldehyde and 2-Naphthaldehyde, respectively. Additionally, gluconolactonase and aldehyde dehydrogenase translated from the genes *gnl* and *aldH* could participate in ring cleavage via Baeyer–Villiger oxidation (Figure 4). This integration could benefit *Sphingomonas* sp. in degrading aromatic compounds and their derivatives. In addition to aromatic compounds, genes related to degrading styrene, atrazine, and other compounds were annotated in the genome of *Sphingomonas* sp. (Figure S2). The catabolic diversity of *Sphingomonas* is much higher than that of other bacteria in terms of the scope and extent of compound degradation. *Sphingomonas* are not obligate bacteria; instead, they are multi-substrate oligotrophic bacteria, which enables them to adapt to a variety of contaminated sites [36].

Recent studies have focused on the physiological and biochemical characteristics of *Sphingomonas* and their ecological functions. To date, the NCBI database has sequenced more than 335 *Sphingomonas*, yielding 22 complete genomes [37]. The complete genomes ranged in size from 2.8 Mb to 6.5 Mb and GC content from 65% to 66.6%. Our study enriches the understanding of the functional potential of *Sphingomonas* in contaminated environments.

**Figure 5:**
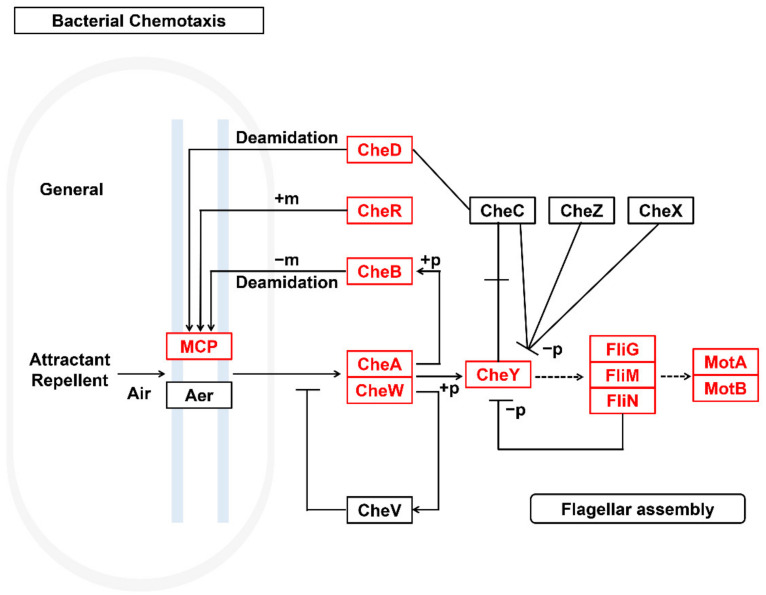
*Sphingomonas* sp. bacterial chemotaxis pathway.

**Figure 6:**
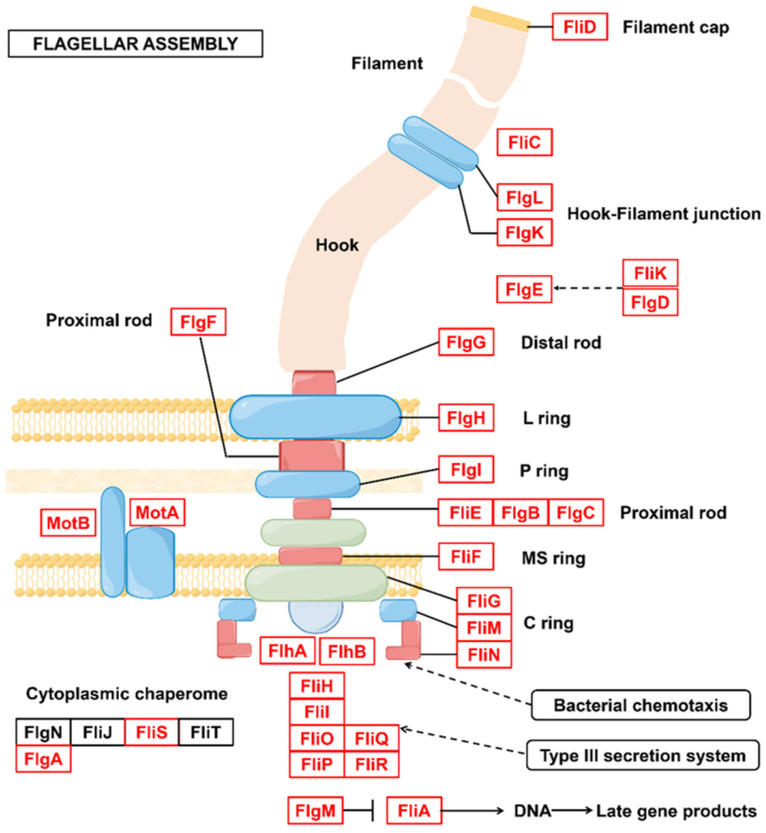
*Sphingomonas* sp. Flagellar assembly pathway.

Complete and abundant bacterial chemotaxis genes (Figure 5) and flagellar assembly genes (Figure 6) were detected in the genome of *Sphingomonas* sp. A total of 80 genes related to flagellar assembly were detected in the *Sphingomonas* sp. genome. Thirty-six genes related to bacterial chemotaxis were detected. Flagellar assembly and chemotaxis genes could help them adapt to harsh PAH-contaminated habitats, access resources, and gain ecological niche advantage.

There are many factors that affect the biodegradation rate of pollutants, and adequate contact between microorganisms and pollutants is one of the important factors. Bacterial chemotaxis is the movement of cells with flagellar, fimbriae, or sliding structures in response to chemical gradients. Chemotaxis enhances bacterial motility to localize and degrade compounds [38]. Chemotaxis is an important physiological adaptation that enables many motile bacteria to position themselves for better niche adaptation, improve the bioavailability of pollutants, and increase the rate of biodegradation [39]. To date, published reports have employed a chemotactic sorting-based microfluidic SlipChip, and demonstrated that chemotaxis to biodegradable pollutants enhanced their bioavailability and increased their bioavailability and biodegradation rate, which also provides new insights for screening effective pollutant-degrading bacteria [40].

## 4. Conclusions

In this study, soil samples at three depths ranging of 0–100 cm were selected from a PAH-contaminated coking site at 70-day intervals. We explored the migration and degradation process of 16 PAHs in the site’s soil. We combined molecular ecology network analysis, metagenomics, and genome assembly to identify the key microorganisms that play important ecological functions in the degradation process and analyze their strategies to maintain a competitive advantage. The results showed that 15 PAHs were identified in the site’s soil. After 70 days, a total of 15.78 mg/kg of PAHs in the 0–100 cm site soil layer naturally decayed, and a total of 13.33 mg/kg PAHs migrated from the 30–100 cm bottom-layer soil to the 10–30 cm middle-layer and 0–10 cm top-layer soils. The total PAH concentration in the top-layer soil rose from not detected to 7.02 mg/kg. *Sphingomonas* had the highest average relative abundance of 5.98% among the 507 genera. Its spatial distribution pattern and temporal change dynamics were consistent with those of PAHs. *Sphingomonas*, which occupied a niche advantage, persisted in the site’s soil and played a crucial role in maintaining the community’s structure. Therefore, *Sphingomonas* was the dominant, core, and keystone microorganism in the site’s soil. On this basis, we successfully assembled the genome of *Sphingomonas* sp. with a completeness of 98.57% and size of 2.52 Mb. Functional genes mediating naphthalene and benzo[a]pyrene degradation were found in the genome sequence. *Sphingomonas* sp. integrated a wide range of aromatic metabolic pathways, including the meta-cleavage of catechol, the dihydroxylation and meta-cleavage of aromatic rings mediated by dioxygenase and dehydrogenase, and the ring cleavage via Baeyer–Villiger oxidation. Complete and abundant bacterial chemotaxis genes and flagellar assembly genes were detected in the genome of *Sphingomonas* sp. The flagellar assembly and chemotaxis genes could help them adapt to the harsh habitat, metabolize aromatic compounds and various substrates, such as styrene and atrazine, and maintain niche advantage. Our results enriched the understanding of microorganisms that play key ecological functions in PAH-contaminated sites, further illustrated the important contribution and great potential of *Sphingomonas* in PAH degradation, and have reference value for the restoration of PAH-contaminated areas, such as smelting sites.

## Data Availability

The data that support the findings of this study are available from the corresponding author (B.H.) upon reasonable request.

## Supplementary Materials

The following supporting information can be downloaded at: https://www.mdpi.com/article/10.3390/microorganisms10061109/s1. Table S1. Detection limit of the 16 PAHs test; Table S2. Relative abundance of 64 core species (T: 0–10 cm; M: 10–30 cm; B: 30–100 cm). Based on the criteria of average relative abundance of >0.1% and occurrence frequency of >80%, the core species in the site’s soil were screened; Table S3. Results of read-filtering in sequencing data analysis (T: 0–10 cm; M: 10–30 cm; B: 30–100 cm); Table S4. Alpha biodiversity indicators of microbes at the coking site (T: 0–10 cm; M: 10–30 cm; B: 30–100 cm); Table S5. Concentrations of the PCR products of DNA from soil samples on Day 0; Table S6. Concentrations of the PCR products of DNA from soil samples on Day 70; Figure S1. Sphingomonas sp. genome circle map; Figure S2. Sphingomonas sp. metabolic pathways for the degradation of styrene and atrazine; Figure S3. Gel electrophoresis images of the PCR products of DNA from soil samples on Day 0; Figure S4. Gel electrophoresis images of the PCR products of DNA from soil samples on Day 70.
