# Supplementary material for: Sphingomonas Relies on Chemotaxis to Degrade Polycyclic Aromatic Hydrocarbons and Maintain Dominance in Coking Sites
Source: Microorganisms. 2022 May 27;10(6):1109. doi: 10.3390/microorganisms10061109 (PMC9229013; doi:10.3390/microorganisms10061109)
Supplement: Supplementary file 1 [file microorganisms-10-01109-s001.zip › microorganisms-1685185-supplementary.pdf]

microorganisms

Article

Sphingomonas relies on chemotaxis to degrade polycyclic aromatic hydrocarbons and maintain dominance in coking sites

Meng Zhou [1], Zishu Liu [1], Jiaqi Wang [1] and Yuxiang Zhao [1]and Baolan Hu [1,2,3,*]

Supplementary Information

Table S1. Detection limit of the 16 PAHs test.

Test items	NAP	ACY	ACE	FLO	PHE	ANT	FLA	PYR
Detection limt(mg/kg)	0.09	0.1	0.1	0.08	0.1	0.1	0.2	0.1
Test items	BaA	BaP	BbF	BkF	CHR	DhA	IcP	BgP
Detection limt(mg/kg)	0.1	0.1	0.2	0.1	0.1	0.1	0.1	0.1

Abbreviations: NAP-naphthalene; ACY-acenaphthylene; ACE-Acenaphthene; FLO-fluorene; PHE-phenanthrene; ANT-anthracene; FLA-fluoranthene; PYR-pyrene; BaA-benzo[a]anthracene; BaP-benzo[a]pyrene; BbF-benzo[b]fluoranthene; BkF-benzo[k]fluoranthene; CHR-chrysene; DhA-dibenzo[a,h]anthracene; IcP-indeno[1,2,3-cd]pyrene; BgP-benzo[g,h,i]perylene.

Table S2. Relative abundance of 64 core species (T: 0–10 cm; M: 10–30 cm; B: 30–100 cm). Based on the criteria of average relative abundance of >0.1% and occurrence frequency of >80%, the core species in the site's soil were screened.

species	Relative Abundance/%					
	Day 0-T	Day 0-M	Day 0-B	Day 70-T	Day 70-M	Day 70-B
Sphingomonas	1.04	2.33	3.12	8.89	13.78	6.73
MND1	2.95	2.50	2.35	2.42	2.89	7.04
Ellin6067	1.11	0.64	0.48	5.81	5.28	3.93
RB41	4.90	3.29	2.17	1.71	0.29	1.79
Nitrospira	0.59	2.37	1.15	0.53	0.91	0.96
Dongia	1.19	1.53	1.73	0.52	0.54	0.92
Haliangium	0.90	1.24	0.52	0.89	0.68	0.84
Steroidobacter	0.82	0.65	0.96	0.52	0.29	1.73
Bryobacter	1.31	0.89	0.42	0.67	1.26	0.40

Ramlibacter	0.11	0.54	0.08	1.36	1.75	1.00
Bradyrhizobium	0.85	0.84	0.54	0.63	0.81	0.48
Terrimonas	0.40	0.60	0.20	0.72	0.78	0.32
IS-44	0.17	0.48	0.55	0.63	0.33	0.40
Sulfurifustis	0.03	0.10	2.20	0.03	0.02	0.13
Reyranella	0.51	0.41	0.26	0.33	0.58	0.34
Pedomicrobium	0.58	0.60	0.81	0.05	0.10	0.20
Gaiella	0.42	0.39	0.35	0.22	0.29	0.65
Rhodoplanes	0.39	0.30	0.04	0.57	0.49	0.44
Acidibacter	0.99	0.36	0.33	0.21	0.07	0.23
Flavitalea	0.07	0.31	0.04	0.61	0.52	0.63
Pseudarthrobacter	0.02	0.57	0.20	0.39	0.49	0.41
Gemmatimonas	0.18	0.36	0.50	0.45	0.33	0.24
Chthoniobacter	0.98	0.19	0.05	0.46	0.27	0.09
Phenylobacterium	0.17	0.15	0.17	0.75	0.53	0.21
Streptomyces	0.09	0.18	0.25	0.42	0.48	0.44
Polycyclovorans	0.10	0.51	0.77	0.05	0.07	0.32
Candidatus_Solibacter	0.46	0.43	0.13	0.20	0.36	0.16
Subgroup_10	1.21	0.23	0.23	0.02	0.02	0.02
Flavobacterium	0.31	1.01	0.12	0.17	0.05	0.05
Pseudomonas	0.24	0.91	0.30	0.08	0.06	0.09
SWB02	0.36	0.31	0.79	0.02	0.07	0.04
Niastella	0.19	0.14	0.07	0.53	0.52	0.02
Bacillus	0.03	0.09	0.45	0.26	0.19	0.39
Flavisolibacter	0.05	0.10	0.03	0.57	0.58	0.08
Altererythrobacter	0.17	0.11	0.18	0.22	0.40	0.28
Nordella	0.24	0.54	0.18	0.10	0.17	0.11
Lysobacter	0.20	0.13	0.06	0.15	0.18	0.53

Hyphomicrobium	0.15	0.20	0.30	0.11	0.15	0.22
Candidatus_Udaeobacter	0.51	0.09	0.02	0.23	0.13	0.13
Iamia	0.15	0.16	0.32	0.07	0.08	0.31
Stenotrophobacter	0.72	0.17	0.17	0.01	0.00	0.01
Opitutus	0.10	0.08	0.03	0.29	0.35	0.20
Cupriavidus	0.02	0.19	0.05	0.21	0.41	0.17
Caenimonas	0.01	0.04	0.03	0.14	0.16	0.61
Ferruginibacter	0.09	0.14	0.02	0.38	0.31	0.03
Thiobacillus	0.00	0.01	0.91	0.00	0.00	0.01
Massilia	0.03	0.11	0.09	0.24	0.23	0.23
Mesorhizobium	0.06	0.07	0.06	0.24	0.38	0.10
mle1-7	0.28	0.33	0.10	0.06	0.01	0.11
Novosphingobium	0.03	0.06	0.04	0.37	0.27	0.12
SM1A02	0.44	0.11	0.08	0.10	0.05	0.09
Hirschia	0.38	0.23	0.14	0.05	0.02	0.04
Mycobacterium	0.17	0.14	0.27	0.10	0.04	0.07
Sphingobium	0.01	0.12	0.16	0.20	0.20	0.04
Rhizobium	0.08	0.11	0.07	0.17	0.23	0.07
Ilumatobacter	0.15	0.17	0.14	0.11	0.05	0.09
Bdellovibrio	0.14	0.19	0.09	0.07	0.06	0.15
Nocardioides	0.14	0.25	0.06	0.06	0.07	0.12
Pseudorhodoplanes	0.18	0.12	0.12	0.09	0.10	0.07
PAUC26f	0.01	0.05	0.51	0.02	0.06	0.02
AKYG587	0.50	0.04	0.11	0.01	0.00	0.02
Anaeromyxobacter	0.00	0.03	0.43	0.04	0.01	0.11
Pseudolabrys	0.09	0.08	0.12	0.09	0.07	0.17
Comamonas	0.28	0.25	0.05	0.02	0.01	0.01

Table S3. Results of read-filtering in sequencing data analysis (T: 0–10 cm; M: 10–30 cm; B: 30–100 cm).

	Day 0-T	Day 0-M	Day 0-B	Day 70-T	Day 70-M	Day 70-B
Number of reads	121840	130387	126842	83998	83954	84000
Filtered reads	121481	129997	126396	83809	83782	83802
Unique reads after filtering	106742	98570	115603	77105	67642	74988
Chimeric reads	-	-	-	-	-	-
Unique chimeric reads	-	-	-	-	-	-
Reads in OTUs	55184	54954	56180	42875	42739	42321

Table S4. Alpha biodiversity indicators of microbes at the coking site (T: 0–10 cm; M: 10–30 cm; B: 30–100 cm).

Alpha-biodiversity	Day 0-T	Day 0-M	Day 0-B	Day 70-T	Day 70-M	Day 70-B
chao1	3453.2	3959.3	4001.3	2572.4	2374.7	2660.5
shannon	9.36	9.91	9.82	8.56	8.38	8.95

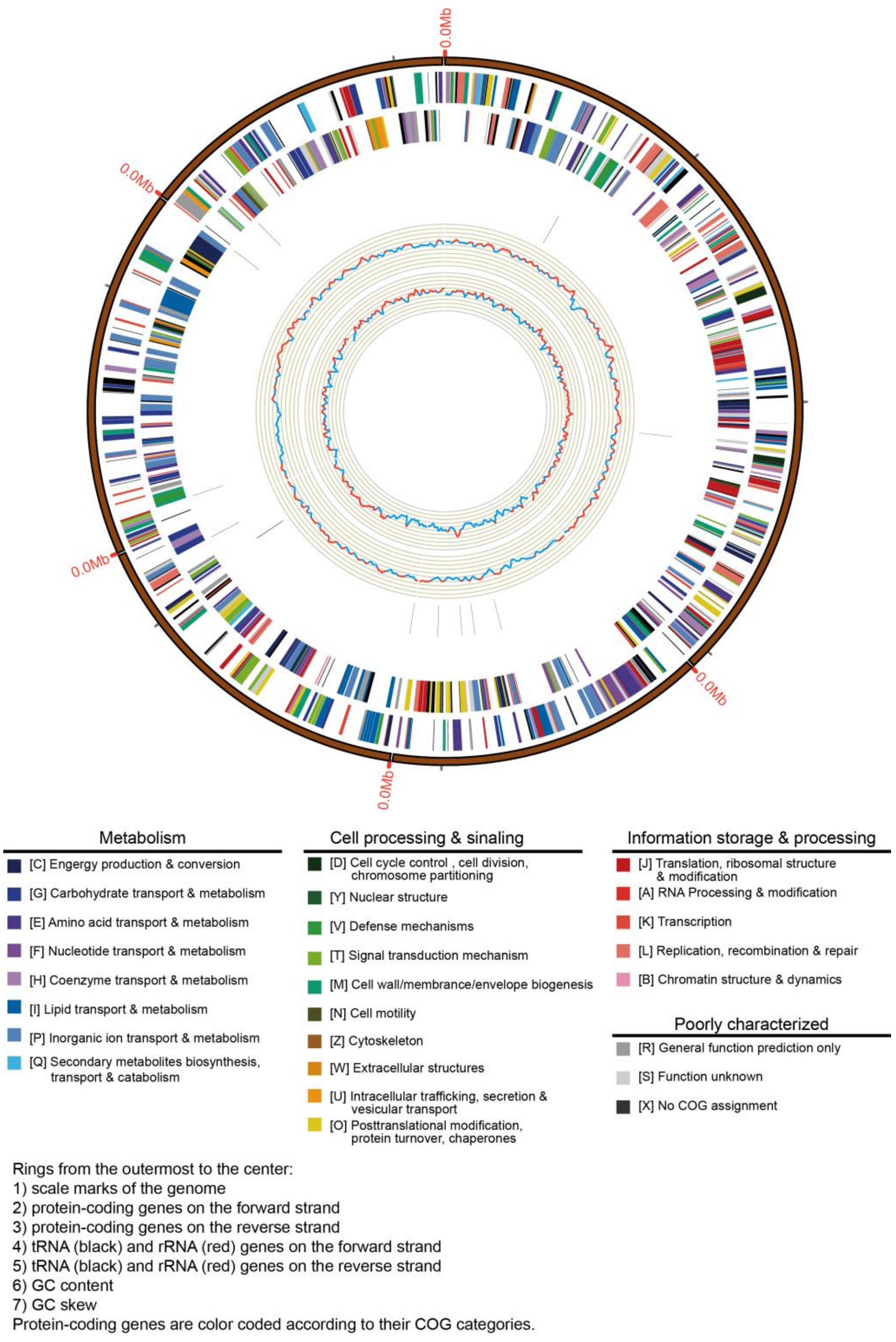

Figure S1. *Sphingomonas* sp. genome circle map.

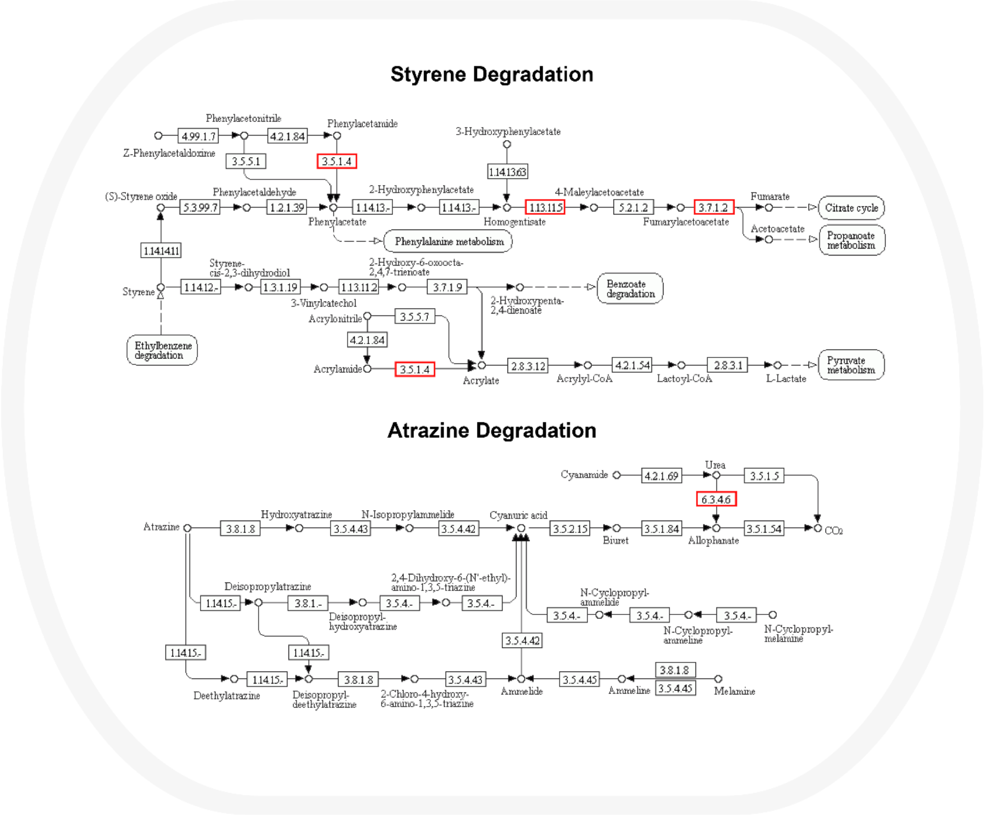

Figure S2. *Sphingomonas* sp. metabolic pathways for the degradation of styrene and atrazine.

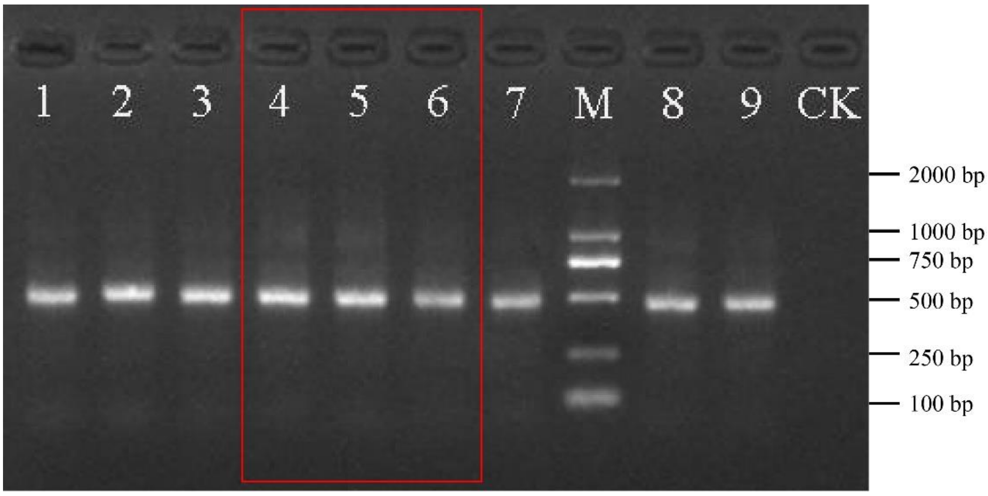

Figure S3. Gel electrophoresis images of the PCR products of DNA from soil samples on Day 0.

Table S5. Concentrations of the PCR products of DNA from soil samples on Day 0.

Sample	Lane	Concentration/(ng/μL)
Day 0-T	4	30.78
Day 0-M	5	28.81
Day 0-B	6	21.74

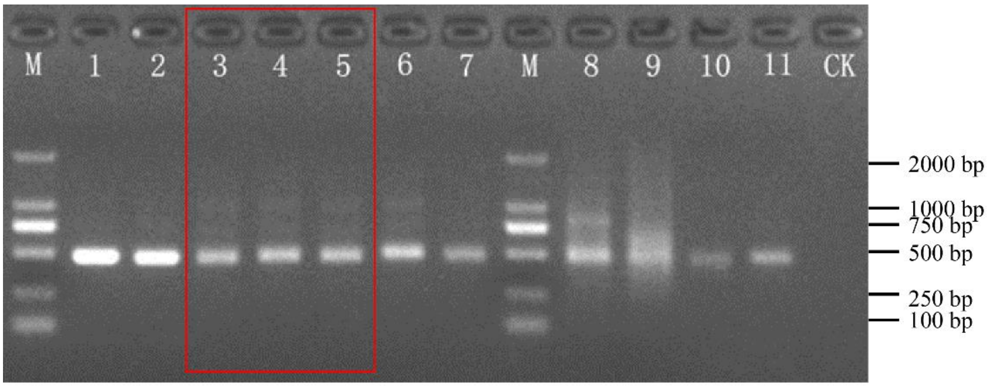

Figure S4. Gel electrophoresis images of the PCR products of DNA from soil samples on Day 70.

Table S6. Concentrations of the PCR products of DNA from soil samples on Day 70.

Sample	Lane	Concentration/(ng/μL)
Day 70-T	3	21.78
Day 70-M	4	23.51
Day 70-B	5	22.98
